# Ionic liquids for the passive sampling of sulfonamides from water—applicability and selectivity study

**DOI:** 10.1007/s00216-017-0342-6

**Published:** 2017-04-11

**Authors:** Hanna Męczykowska, Paulina Kobylis, Piotr Stepnowski, Magda Caban

**Affiliations:** Institute for Environmental and Human Health Protection, Faculty of Chemistry, University of Gdansk, ul. Wita Stwosza 63, 80-308 Gdańsk, Poland

**Keywords:** Ionic liquid, Passive sampling, PASSIL, Pharmaceuticals, Sulfonamides, Water monitoring

## Abstract

Ionic liquids (ILs) are new-generation, non-volatile solvents which are designable, and their structure may be specifically adjusted to the current application needs. Therefore, it is possible to create and apply ILs which efficiently and selectively extract various analytes from different matrices. It has already been examined that ILs may be applied as receiving phases in passive sampling for the long-term water monitoring of PAHs and pharmaceuticals in water. In this paper, the concept of passive sampling with ILs (PASSIL applied as receiving phases) was continued and developed using phosphonium-, imidazolium-, and morpholinium-cation-based ILs. The target group of analytes was pharmaceuticals which represent one of the most common categories of water contaminants. Fourteen-day-long extractions using various ILs were performed in stirred conditions at a constant temperature (20 °C). The best extraction efficiency was achieved for trihexyl(tetradecyl)phosphonium dicyanamide ([P666-14][N(CN)_2_]). For this preliminary calibration, the sampling rates were calculated for each sulfonamide. Once again, selectivity was observed in passive sampling using [P666-14][N(CN)_2_]. Therefore, PASSIL is seen as a very promising method for pharmaceutical monitoring in water.

**Electronic supplementary material:**

The online version of this article (doi:10.1007/s00216-017-0342-6) contains supplementary material, which is available to authorized users.

## Introduction

The monitoring of pharmaceuticals is one of the fastest developing issues in current analytical chemistry in the field of environmental research. Pharmaceuticals are considered to be emerging pollutants due to their increasing consumption (considering expenditure and the volume or quantity of medicines consumed) [1], low removal efficiency in wastewater treatment plants [2], and negative impact on the life of water organisms [3]. For these reasons, data among various water contaminants should also be gathered for the monitoring of pharmaceuticals, according to the EU [4].

Sulfonamides (SAs) are one of the most widely used antibiotics [5, 6]. They are the second most commonly applied veterinary medicines in the European Union. These compounds are not completely metabolized; therefore, unmetabolized SAs and their metabolites are released into the environment [7]. Several studies confirmed that sulfonamides enter the environment by wastewater effluents [8] and can stream along with the surface or groundwater or remain in the soil [9]. Sulfonamides, like other pharmaceuticals, are monitored in water environments using various methods, like grab sampling [10–13], solid-phase extraction [14], the radioimmunoassay technique [15], micro-solid-phase extraction [16], or passive sampling (e.g., the polar organic chemical integrative sampler—POCIS technique) [17–19].

Passive sampling allows time-weighted average concentrations (TWACs) to be received, which are independent from random changes of environmental conditions [20–24]. Therefore, it is suitable for the long-term monitoring of contamination levels. Passive sampling techniques differ according to the receiving phase type. The most popular passive samplers contain divinylbenzene–*N*-vinylpyrrolidone copolymer (DVB–NVP) in pharmaceutical-POCIS, and modified silica (C18) in pesticide-POCIS and Chemcatcher [25–28].

Recently, we found that ionic liquids (ILs) may be applied as the liquid receiving phase in the passive sampling of PAHs [29] and selected pharmaceuticals [30]. ILs contain large, bulky, organic cations and significantly smaller organic or inorganic anions. Due to their unique structure, these salts are liquid in room temperature. Ionic liquids are also characterized by chemical and thermal stability and very good solvent properties, which are useful for passive sampling. The innovative technique of passive sampling was called *pas*sive *s*ampling with *i*onic *l*iquids (PASSIL). In the current study, passive sampling was performed for eight sulfonamides using phosphonium-, imidazolium-, and morpholinium-cation-based ionic liquids. The aim of this study was to find the proper ionic liquid which may be applied as the receiving phase for the passive sampling of these common water pollutants. It is expected that the PASSIL technique will allow for the dynamic and efficient extraction of pharmaceuticals from an aqueous medium.

## Materials and methods

### Chemicals

**Fig. 1:**
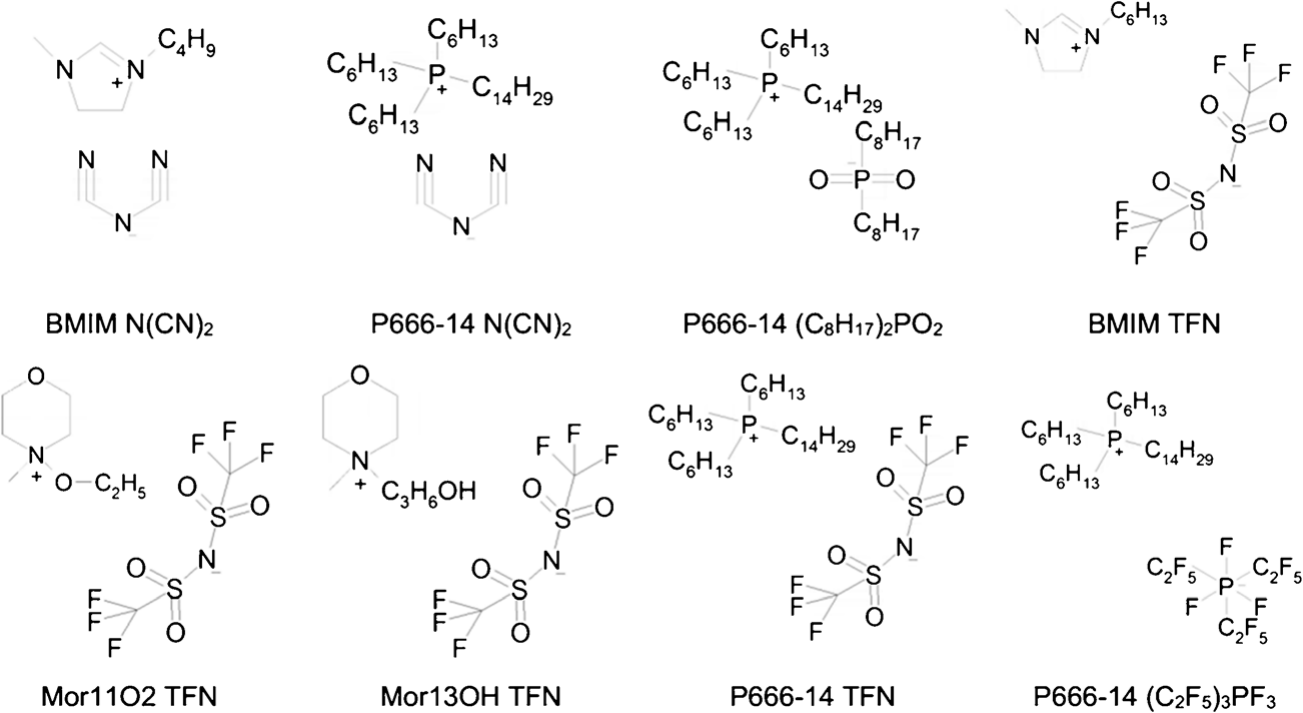
Ionic liquids applied during this study

ILs were obtained from the University of Bremen (Germany) and Sigma-Aldrich (Germany) at a purity of ≥95%. The ionic liquids applied were (Fig. 1) trihexyl(tetradecyl)phosphonium dicyanamide ([P666-14][N(CN)_2_]), trihexyl(tetradecyl)phosphonium tri(pentafluoroethyl)trifluorophosphate ([P666-14][(C_2_F_5_)_3_PF_3_]), trihexyl(tetradecyl)phosphonium dioctylphosphate [P666-14][(C_8_H_17_)_2_PO_2_], trihexyl(tetradecyl)phosphonium bis(trifluoromethylsulfonyl)imide [P666-14][TFN], 4-(etoxy)-4-methylmorpholinium bis(trifluoromethylsulfonyl)imide ([Mor13OH][TFN]), 4-(hydroxypropyl)-4-methylmorpholinium bis(trifluoromethylsulfonyl)imide ([Mor11O2][TFN]), and 1-butyl-3-methylimidazolium dicyanamide ([BMIm][N(CN)_2_]).

**Fig. 2:**
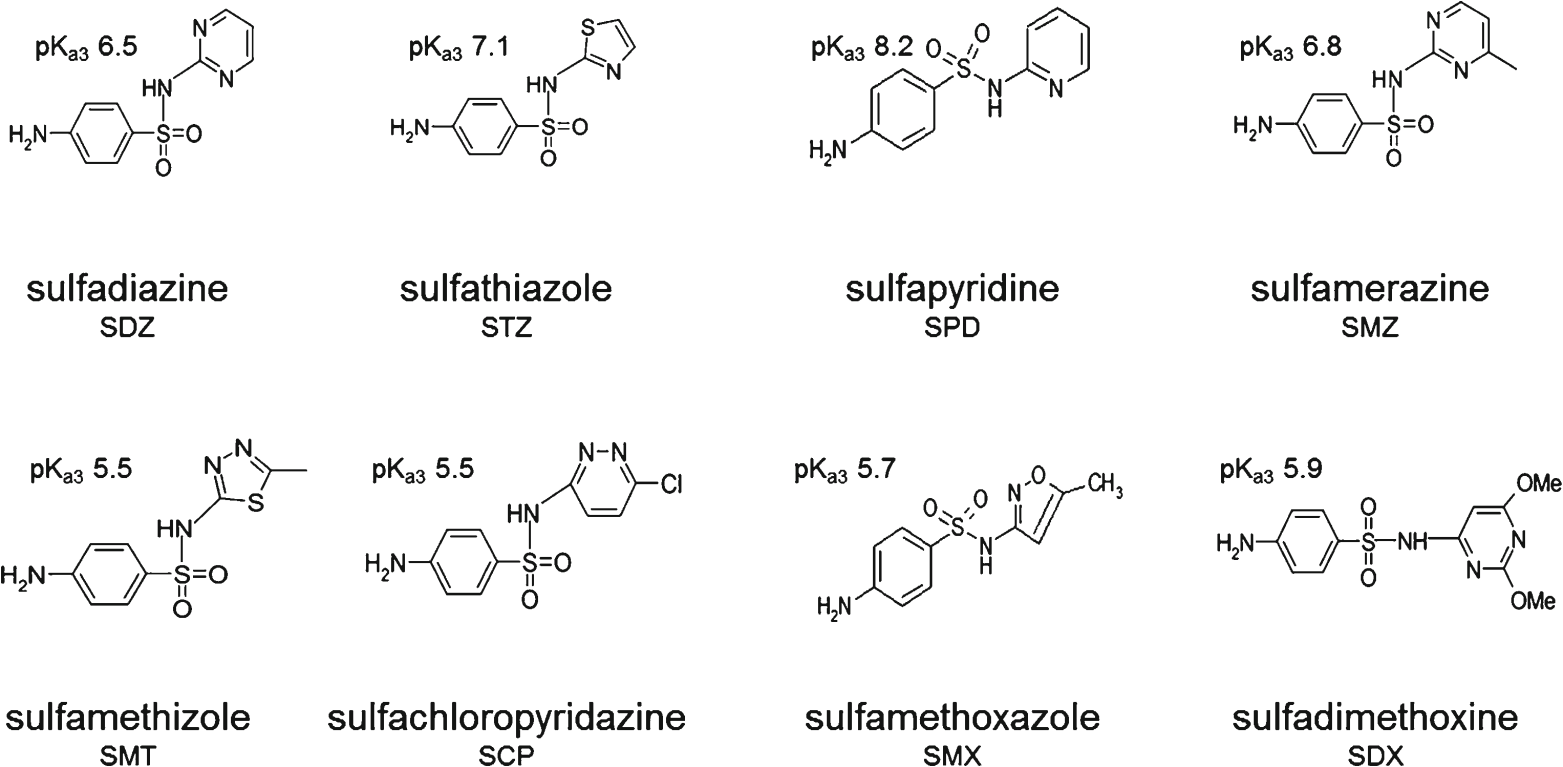
Sulfonamides applied as target analytes during PASSIL

Standards of SAs (Fig. 2), sulfathiazole (STZ), sulfamethiazole (SMT), sulfachloropyridazine (SCP), sulfamethoxazole (SMX), sulfadimethoxine (SDX), sulfadiazine (SDZ), sulfapyridine (SPD), and sulfamerazine (SMZ), were also purchased from Sigma-Aldrich (Germany). HPLC solvents, *n*-octanol, and ammonium acetate were obtained from POCH (Poland).

### Methods

#### Laboratory calibration using ultrapure water

Passive sampling experiments were conducted analogously to previous studies [30]. The passive dosimeter was made of plexiglass and contained two polyethersulfone (PES) membranes with their inner sides covered with the receiving phase—ionic liquid (separately for each of the seven tested ILs) of a total mass equal to 200 mg. Nylon (Phenomenex) and Teflon (Merck) membranes were also tested. The donor phase was 100 mL of water solution with an initial sulfonamide concentration of 2 μg mL^−1^. The experimental setup was kept away from light radiation and at a constant temperature (20 °C). Each extraction lasted 14 days and the samples (1 mL) of the donor solution were collected every 2 days. The solution was stirred using a magnetic stirrer (1000 rpm), which was not renewed. After the experiment, the receiving phase was dissolved using 50 mL of acetonitrile. All the obtained samples (both from the donor and receiving phases) were analyzed by HPLC with a diode-array detector (DAD, Schimadzu, 272 nm). The analysis was performed using a Gemini 5-μm C18 column (Phenomenex). The injection volume was 10 μL. The flow rate was 1.5 mL min^−1^. Acetonitrile (ACN) was used as the organic component of the mobile phase, and the aqueous component was an acetate buffer (5 mmol L^−1^ CH_3_COONH_4_, pH = 4) with 5% of ACN. The mobile phase gradient was applied. The recovery of the receiving phase, the extraction efficiency, and the sampling rate (for [P666-14][N(CN)_2_]) were calculated for each analyte according to the equations presented in [30]. Moreover, micrographs using a HITACHI S-3400N scanning electron microscope (SEM) were taken to study the morphological appearance of the membranes covered with an ionic liquid [P666-14][N(CN)_2_].

#### Laboratory calibration using environmental water samples

Two types of environmental water samples were collected: surface water and secondary effluent. The first one was collected from the Oliwski Stream, and the second one was obtained from a wastewater treatment plant “Wschód” in Gdańsk. All collected samples were stored at 4 °C and kept out of light radiation. Before the extraction, water and effluent samples were filtrated using a glass filtration set (Sartorius, Germany) with glass fiber filters.

Four extractions of pharmaceuticals from the surface water were conducted, two in 1 L of raw water and another two in 1 L of water spiked with eight SAs to obtain an initial concentration of 2 μg L^−1^. Simultaneously, another four extractions of sulfonamides from sewage water were performed, likewise in raw and spiked (2 μg per 100 mL) water of total volume 100 mL each. Passive dosimeters were prepared just like during the calibration in ultrapure water. Each experiment lasted 7 days and samples (0.5 mL) were collected every 24 h. HPLC analysis was conducted analogously to previous PASSIL experiments.

#### Determining log*K*_OW_ and log*D*_OW_ values

The octanol–water coefficient (*K*_OW_) can be defined as the ratio of the compound concentration between an organic solvent (usually *n*-octanol) saturated with water and an aqueous phase (e.g., water saturated with *n*-octanol) [31]. The coefficient is usually used in a logarithmic form (log*K*_OW_). However, this value does not vary in the context of various forms of ionization of the analyte. Therefore, to solve this issue, the *K*_OW_ may be additionally multiplied by the *f*_n_ factor (fraction of neutral molecule). The received value is *D*_OW_ (pH-adjusted octanol–water distribution coefficient), also applied in the logarithmic form (log*D*_OW_). *D*_OW_ may be defined as the ratio of the concentrations of both the ionized and unionized species of the compound in the organic and aqueous phases at a specific pH value. During this study, the log*D*_OW_ values were determined experimentally for all tested antibiotics. Solutions of each sulfonamide were prepared separately at a concentration of 100 μg mL^−1^ in methanol. The obtained solutions were diluted with ultrapure water (pH 5.6) previously saturated with octanol to obtain a concentration of 5 μg mL^−1^. Equal volumes of the prepared solutions and octanol saturated with water were shaken and allowed to settle at room temperature in order to separate two clear layers. Then, 0.5 mL of the water layer of each sample was taken and analyzed by HPLC/DAD (272 nm). On the basis of this experiment, the log*K*_OW_ and log*D*_OW_ values were calculated [32].

## Results and discussion

### Stability of ILs inside the PASSIL dosimeter

**Fig. 3:**
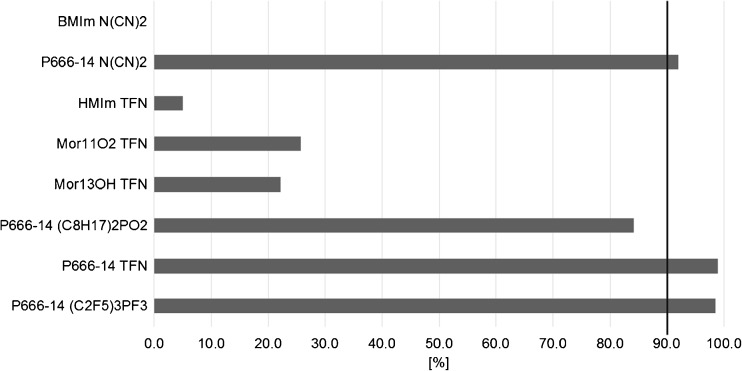
Recovery (%) of ionic liquids applied as receiving phases in PASSIL

The basic parameter of an applied ionic liquid is its physical stability inside the homemade dosimeter expressed by the IL recovery (%) calculated from the difference between ionic liquid masses before and after the passive sampling. The highest recovery (more than 90%) of ionic liquids was achieved by phosphonium-cation-based ILs and PES membranes (Fig. 3). The recoveries were 98.9% for [P666-14][TFN], 98.5% for [P666-14][(C_2_F_5_)_3_PF_3_], and 92.4% for [P666-14][N(CN)_2_]. The type of cation seems to have a bigger impact on the stability of the tested ILs, which was found after a comparison of the results for bis(trifluoromethylsulfonyl)imide (TFN)-based ILs.

Among all the examined ionic liquids, trihexyl(tetradecyl)phosphonium dicyanamide [P666-14][N(CN)_2_] was characterized as having the third best recovery, although it allowed for the best efficiency of the extraction of sulfonamides. Also, [P666-14][N(CN)_2_] contains no fluorine atoms; therefore, it is less toxic for living organisms than the other two ionic liquids, which contain fluorinated anions [33]. Thereby, during further experiments, [P666-14][N(CN)_2_] was applied.

#### Nylon, PTFE, and PES membrane application

**Fig. 4:**
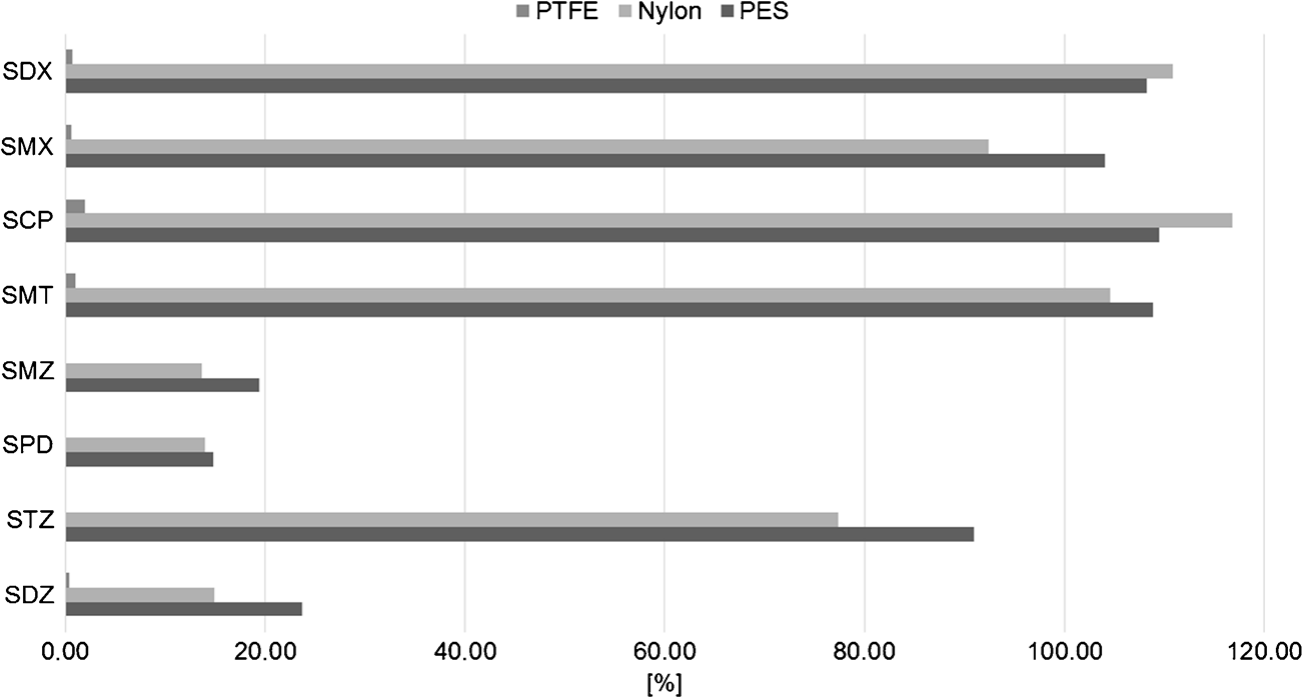
Relation between the efficiency of the passive sampling extraction of sulfonamides with [P666-14][N(CN)_2_] as the receiving phase, and the membrane types (PES, nylon, or PTFE)

The next step was to examine the influence of membrane types for the extraction of SAs using the previously selected ionic liquid. It was confirmed that the best membranes are nylon and PES (Fig. 4). The application of polytetrafluoroethylene (PTFE) membranes results in a very low concentration or lack of analytes in the receiving phase.

**Fig. 5:**
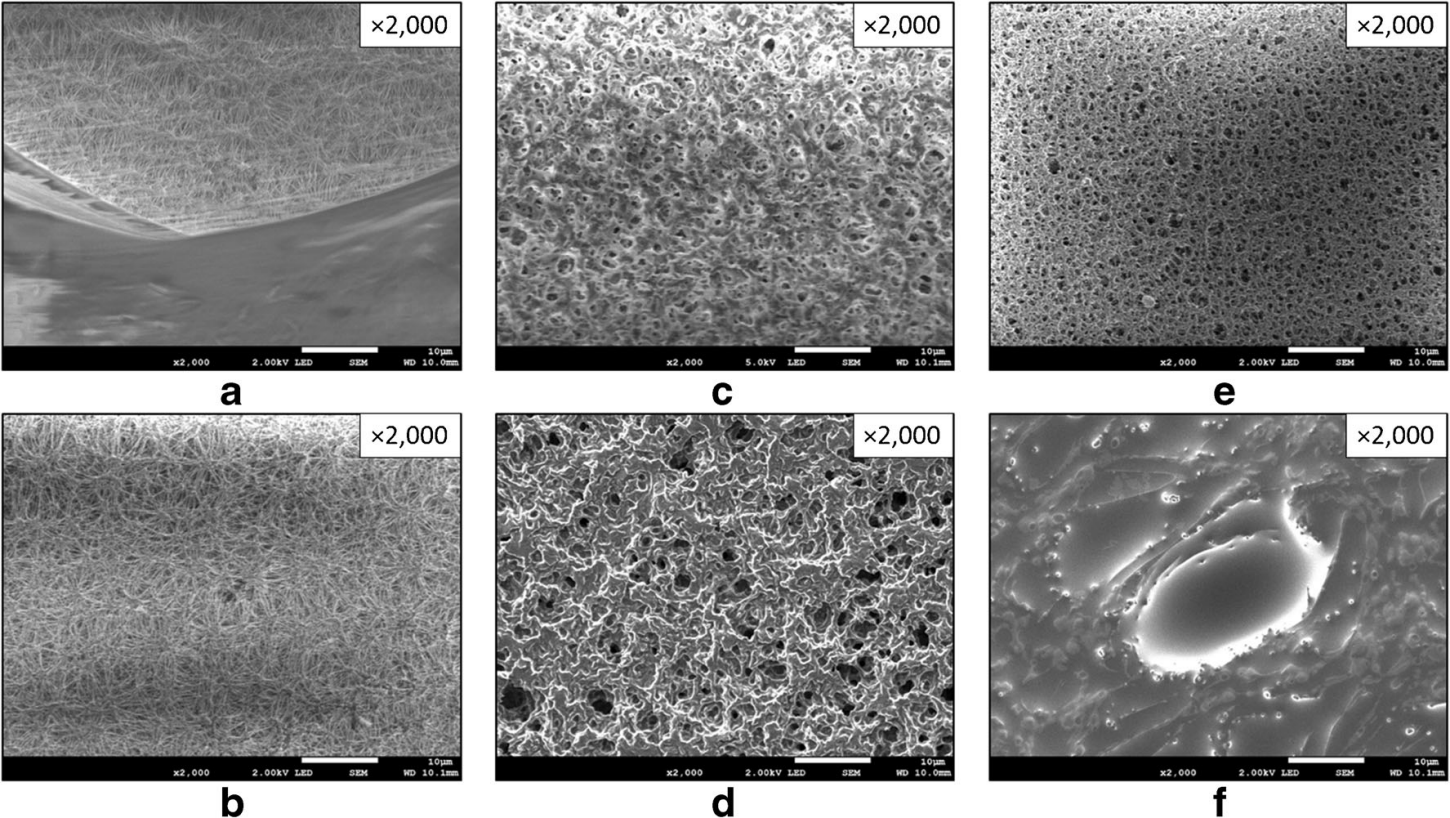
SEM micrographs of PTFE membranes uncovered (**a**) and covered (**b**) with an ionic liquid, nylon membranes uncovered (**c**) and covered (**d**) with an ionic liquid, and PES membranes uncovered (**e**) and covered (**f**) with an ionic liquid [P666-14][N(CN)_2_]

The applicability of nylon and PES membranes for PASSIL was confirmed also by the SEM micrographs (Fig. 5). However, the coating with an ionic liquid was the best for the polyethersulfone (PES) membrane—almost all of the pores were filled with the acceptor phase (Fig. 5). For nylon, the membrane coating was visibly smaller. For PTFE, almost no coating was observed. The hydrophobicity of PES membranes is relatively small compared to that of the other tested types, but the covering with hydrophobic ILs was satisfying.

Control experiments (membranes without a receiving phase) were performed. PES membranes do not significantly adsorb the analytes on their surface (≤12%), while other membranes participate in the extraction of analytes. Finally, the membrane selected for PASSIL experiments was PES. The same membrane type is also used in the POCIS technique [34].

**Fig. 6:**
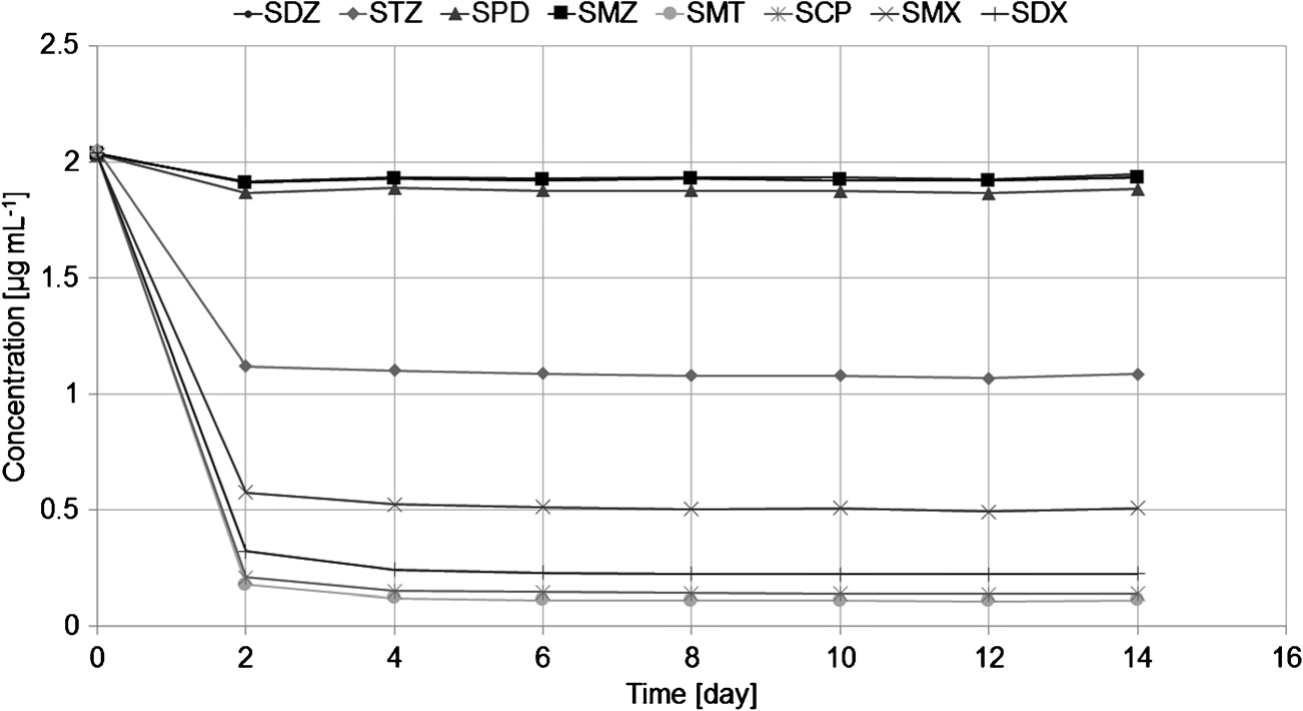
The concentration of sulfonamides in a water solution during passive sampling with PES membranes and [P666-14][N(CN)_2_] applied as a receiving phase (experiment conducted in triplicate)

Figure 6 shows the change in the concentration of target SAs in a water phase in 14-day controlled experiments. The most significant analyte concentration drop takes place between the first and second sample collections, similarly to the results from previous investigations for different pharmaceutical types [30]. The drop in concentration (difference between the compound concentration in the donor solution before and after passive sampling) for SMZ and SPD was negligible, while for STZ it was close to 50%. For the rest of the analytes, the drop was more significant (75 to 95%).

#### PASSIL calibration

The sampling rates (see Electronic Supplementary Material (ESM) Table S1) (*R*_s_ defined as the volume of water purified from the analyte per unit of time [L day^−1^]) could be calculated only for analytes whose concentration decrease was greater than 50% (analogously to previous research [30]). The values of sampling rates varied between 0.61 and 0.92 L day^−1^. These numbers are minimum four times higher than analogous results obtained from POCIS experiments [35–37]. This means that the change of the receiving phase from solid to liquid can make passive sampling more efficient. It is very important that the membranes’ pores and surface are covered by the IL, which can directly interact with the analytes. In the POCIS technique, the analytes need to firstly pass through the membrane pores. In the PASSIL technique, the role of the membrane is just to support the liquid receiving phase.

#### PASSIL selectivity

**Fig. 7:**
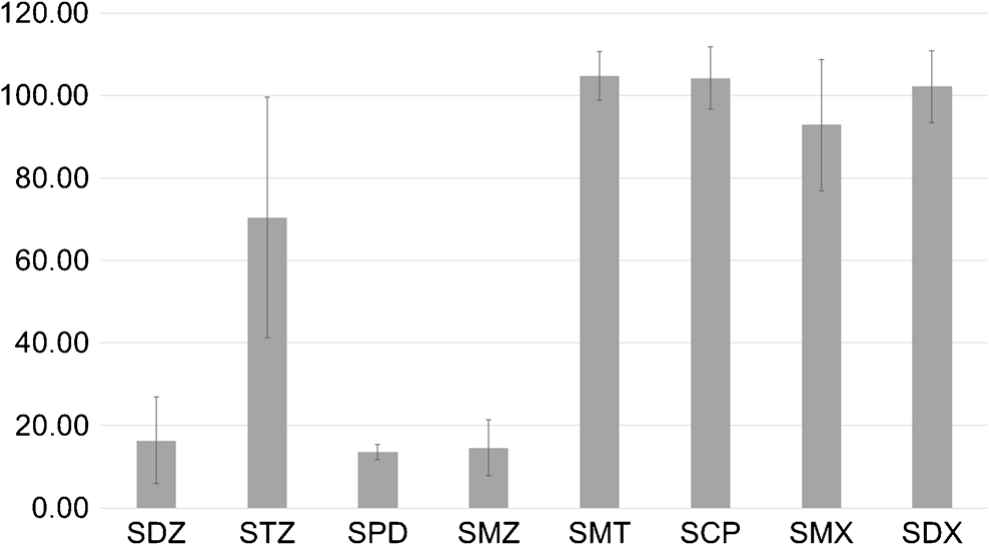
Sulfonamide extraction efficiencies obtained from PASSIL experiments carried out using ultrapure water

The selectivity of PASSIL performed using [P666-14][N(CN)_2_] needs to be discussed. The factors which are responsible for the low extraction efficiency of SDZ, SPN, and SMZ (Fig. 7) are sure to come from the side of the analytes, and the p*K*a and log*K*_OW_ are the most probable factors.

Sulfonamides have three p*K*a values [38], but only two and three are valid for this study. The lowest values of p*K*a_3_ of the analytes are in the range from 5.5 (for SMT and SCP) to 5.9 (for SDX) (Table 1). For these SAs, more than 35.5% of particles are charged negatively (calculated using equations in [39]), and the extraction efficiency is higher than 60%. Also, for sulfathiazole (p*K*a_3_ 7.1), the efficiency is higher than 50%, although its neutral form dominates in the donor solution.

**Table 1:** The ionization degrees, log*K*_OW_, and log*D*_OW_ of sulfonamides (initial pH 5.64)

Parameter	p*K*a_2_	p*K*a_3_	*α*2 [%]	*α*3 [%]	Log*K*_OW_ (exp)	Log*D*_OW pH 5.6_ (exp)
SDZ	1.8	6.5	100.0	12.1	−0.34	−0.39
STZ	2.1	7.1	100.0	3.4	−0.16	−0.17
SPD	2.4	8.2	99.9	0.3	0.04	0.04
SMZ	1.8	6.8	100.0	6.5	0.19	0.16
SMT	1.8	5.5	100.0	58.0	0.01	−0.34
SCP	2.2	5.5	100.0	58.0	0.36	0.00
SMX	1.8	5.7	100.0	46.6	−0.19	−0.44
SDX	2.5	5.9	99.9	35.5	0.59	0.42

**Fig. 8:**
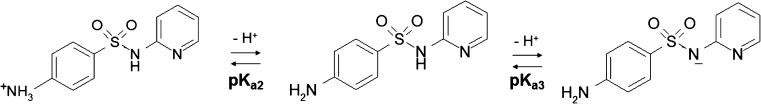
Ionization states of sulfapyridine

For other analytes, whose p*K*a_3_ is higher than the pH value of the donor solution, the negatively charged form is present in less than 13% (for SPD, it is less than 1%) (Fig. 8) and their passive sampling efficiencies are lower than 20%. Thereby, it was assumed that the negative form of the analyte was favored during passive sampling.

In 2011, Li et al. [25] examined the influence of different pH values of the donor solution on the sampling rates for POCIS of estrogens, anti-depressants, β-blockers, and other pharmaceuticals. For acidic pharmaceuticals, the sampling rates decreased along with an increase in the donor solution pH from 3 to 9. The opposite situation was observed for basic analytes. In both cases, the sampling rates were higher when the neutral form of the target compound prevailed. For naproxen, the highest *R*_s_ value (p*K*a 4.20 [40]) was calculated at pH 3, while for nadolol (p*K*a 9.69 [41]), at pH 9. Therefore, it appears that the dependencies during POCIS and PASSIL passive sampling are different, which was assumed.

The sampling rate values are also influenced by the hydrophobicity of the analytes. Lipophilicity is usually measured by the octanol–water (partition) coefficient (*K*_OW_) (“Methods” section). However, how it was already mentioned, for ionizable compounds at a pH at which they are partially ionized, the concentration in both phases is related to the distribution coefficient (*D*). Therefore, for sulfonamides, both coefficients (presented as log*K*_OW_ and log*D*_OW_) were determined experimentally and calculated according to [32]. Generally, the more hydrophobic SAs are extracted more efficiently; however, no direct proportional dependencies between PASSIL efficiency and log*K*_OW_ or log*D*_OW_ values of SAs were noticed.

#### PASSIL from environmental matrices

**Fig. 9:**
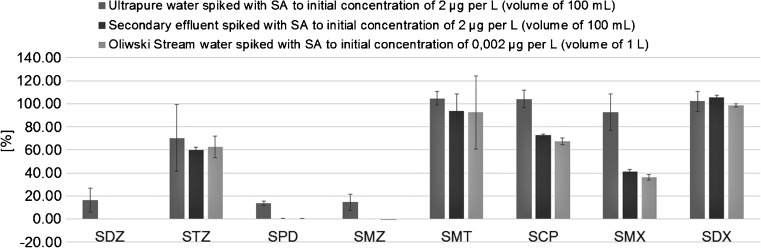
Sulfonamide extraction efficiencies obtained from PASSIL experiments conducted using real environmental water samples

The PASSIL technique has been also used for extraction of sulfonamides from environmental matrices (“Methods” section). The recovery (%) of SAs from water samples collected from Oliwski Stream (Gdansk, Poland) and secondary effluent (Gdansk, Poland) is presented in Fig. 9.

First of all, the change of donor solution from ultrapure water to environmental matrix did not affect the efficiency of PASSIL in the case of STZ, SMT, and SDX. For the rest of the analytes (SDZ, SPD, and SMZ), increased salinity (conductivity of 0.3–0.5 mS cm) and alkalinity (pH 7–8), as was in the case of the use of both environmental matrices, caused a significant decrease of extraction efficiency. The most saline donor solution (water from Oliwski Stream) caused the most decrease.

Moreover, applying different SA concentrations (2 and 0.002 μg L^−1^) also has not influenced the PASSIL efficiency (Fig. 9). Obtained results are compatible with those obtained from POCIS. Togola and Budzinski [42] and DiCarro [43] investigated the initial analyte concentration impact on the *R*_s_ of pharmaceuticals. Both teams have not observed any direct dependencies between the concentration of the compound and its extraction efficiency [44]. Therefore, it is stated that PASSIL passive sampler laboratory calibrations, likewise POCIS, may be performed at higher analyte concentrations (μg L^−1^) than are present in the water environment—pharmaceutical concentrations in river water are usually lower than 500 ng L^−1^.

Additionally, the sampling rates (*R*_s_) for effluent samples were calculated, however only for sulfonamides characterized by a significant (>50%) concentration drop of the donor solution: 0.8 L day^−1^ for STZ and SMT, 0.59 L day^−1^ for SCP, and 0.45 L day^−1^ for SDX. All presented values are higher than those obtained from POCIS calibration experiments held in different environmental conditions [44].

## Conclusions

PASSIL is a novel technique which may be applied for the passive sampling of pharmaceuticals from water. Among the tested imidazolium-, morpholinium-, and phosphonium-cation-based ionic liquids, [P666-14][N(CN)_2_] is characterized by the best acceptor properties. The extraction efficiencies of SAs obtained using PASSIL, both using ultrapure and environmental water, were higher than those received using POCIS (conducted with similar test conditions). As it was already suspected after previous experiments, PASSIL is a selective method of long-term water monitoring. The PASSIL selectivity is most probably based on the ionization degree of the analytes (in this case sulfonamides). The best efficiencies were obtained for more acidic SAs, whose negatively charged form prevails in the donor water solution (pH 5.5 ± 0.1). The PASSIL applicability, mechanism, and variability will be the subject of further research.

## Electronic supplementary material

ESM 1 (PDF 79 kb)
