# Supplementary material for: Ionic liquids for the passive sampling of sulfonamides from water—applicability and selectivity study
Source: Anal Bioanal Chem. 2017 Apr 11;409(16):3951–8. doi: 10.1007/s00216-017-0342-6 (PMC5437200; doi:10.1007/s00216-017-0342-6)
Supplement: Supplementary file 1 — (PDF 79 kb) [file 216_2017_342_MOESM1_ESM.pdf]

Analytical and Bioanalytical Chemistry

Electronic Supplementary Material

Ionic liquids for the passive sampling of sulfonamides from water – applicability and selectivity study

Hanna Męczykowska, Paulina Kobylis, Piotr Stepnowski, Magda Caban

Table S1 Sampling rates [L day^{-1}] values for sulfonamides obtained from PASSIL and POCIS [34–36] experiments

SAs	PASSIL	POCIS	Ref.
SDZ	-	-	-
STZ	0.61±0.11	-	-
SPD	-	0.051	[34]
SMZ	-	0.201±0.06	[35]
SMT	0.92±0.18	0.201±0.06	[36]
SCP	0.92±0.17	0.203±0.06	[35]
SMX	0.79±0.05	0.146±0.06	[35]
SDX	0.88±0.15	0.091	[34]
